# A health concept with a social potential: an interview study with nursing home residents

**DOI:** 10.1186/s12877-020-01731-4

**Published:** 2020-09-04

**Authors:** Sofia Vikström, Helena K. Grönstedt, Tommy Cederholm, Erika Franzén, Åke Seiger, Gerd Faxén-Irving, Anne-Marie Boström

**Affiliations:** 1 Department of Neurobiology, Care Science and Society, Division of Occupational Therapy, Karolinska Institutet, Stockholm, Sweden; 2 Stockholms Sjukhem R&D unit, Stockholm, Sweden; 3 Openlab, Stockholm, Sweden; 4 Allied Health Professionals, Function Area Occupational Therapy & Physiotherapy, Karolinska University Hospital, Stockholm, Sweden; 5 Department of Geriatric Medicine, Uppsala University Hospital, Uppsala, Sweden; 6 Karolinska University Hospital, Theme Aging, Stockholm, Sweden; 7 Department of Public Health and Caring Sciences, Clinical Nutrition and Metabolism, Uppsala University, Uppsala, Sweden; 8 Department of Neurobiology, Care Science and Society, Division of Physiotherapy, Karolinska Institutet, Stockholm, Sweden; 9 Department of Neurobiology, Care Science and Society, Division of Clinical Geriatrics, Karolinska Institutet, Stockholm, Sweden; 10 Department of Neurobiology, Care Science and Society, Division of Nursing, Karolinska Institutet, Stockholm, Sweden

**Keywords:** Older person, Nursing home, Adherence, Health intervention, Sit-to-stand, Oral nutritional supplementation, Interview, Experience, Expectation, Well-being

## Abstract

**Background:**

A qualitative, interview-based study was embedded in a randomized intervention trial, the Older People Exercise and Nutrition (OPEN) study. Participants in the OPEN study were encouraged to conduct sessions of sit-to-stand (STS) exercises combined with Oral Nutritional Supplements (ONS) intake. The aim was to describe the older persons’ perceptions and experiences of being given the daily opportunity to perform the STS exercise and drink ONS.

**Methods:**

In-depth interviews were conducted in six nursing homes with the participants using a semi-structured interview guide. One or two individual interviews were performed with each included participant. Twenty-three NH residents (16 women and 7 men) participated in the qualitative study. Their ages ranged between 76 and 96 years, and their Mini Mental State Examination (MMSE) scored between 8 and 29. The transcribed interviews and field notes written during the visits were analyzed inductively following a constant comparative method described in Grounded Theory.

**Results:**

The exercise and nutritional intervention was described as highly practical by the NH residents, who claimed it also had a social aspect as they felt acknowledged and empowered to engage others in the combined intervention. Experiences of the intervention ranged from neutral to mainly positive and could be sorted into 5 categories: 1. Perceived hopes and expectations, 2. Health-related driving forces, 3. Appreciated daily activities, 4. A concept easy to perform and integrate into daily life, 5. A beneficial health concept for all. The intervention created perceived benefits on various health aspects due to participants feeling energized and stronger. An overall theme was identified as *A health concept with a social potential, as participants feel acknowledged and strong enough to help others*.

**Conclusions:**

The intervention was described by participants as a health concept that could potentially be beneficial for a broader spectrum of NH residents. The findings indicate that health concepts, such as STS/ONS, might contribute to a more meaningful day for older people, even vulnerable NH residents approaching the end of life.

**Trial registration:**

ClinicalTrials.govIdentifier: NCT02702037. Date of trial registration February 26, 2016.

## Background

There is a global tendency to support and encourage older people to remain living in their own homes for longer, prior to nursing home (NH) admission [1, 2]. Consequently, a majority of very old people live in their homes, which are rarely designed to meet their age-related needs. This results in risks for both their mobility and social engagement [3]. A passive lifestyle, combined with increased age at NH admission, results in NH residents having a higher risk of being frail [3].

Later NH admission commonly results in older people having developed multiple chronic conditions, including malnutrition and dementia, before admission [4]. These older persons might therefore be particularly vulnerable to negative health impacts related to institutionalization, such as further reduction in activity levels and social isolation [5]. This has been underscored by a recent Swedish population survey by the Swedish National Board of Health and Welfare (NBHW) [6], where more than half of the respondents, older people in NH, perceived themselves as lonely and inactive. This calls for interventions to address these challenges and promote physical activity, well-being and health.

In line with recommendations for person-centered care, meaningful activities, such as physical activity, should be offered regularly to all older persons in NH [7]. Additionally, the Swedish NBHW [6] states that the care of older people must address their right to live with dignity and perceived good health. The Swedish Social Service Act emphasizes that all NH residents should receive care that meets their individual needs [8]. Studies have shown that many older people’s needs for autonomy and participation in their daily life are unmet when they are admitted to either NH [9] or assisted-living facilities [10].

Physiological factors, such as lower extremity muscle weakness and functional limitations in gait and balance, have been associated with a risk of falling. Fear of falling leads people to adopt a passive, sedentary lifestyle [11], and almost doubles the risk of falls [12]. Although rare, contemporary studies focusing on physiological factors in fall preventive programs for older persons show a positive impact on balance and gait [13] and potentially on mood and autonomy [14]. Positive outcomes have been reported, even in the very old and frail [15], of using a 7-week exercise program [16] or a home-based physical training program [17]. There are indications that simple repetitions of a sit-to-stand activity (STS) can result in increased balance and physical function in NH residents [18].

Another preventive approach is physical exercise in combination with a protein-rich oral nutritional supplement (ONS). Although this is not common, two separate studies using a combination of aerobic physical activity with protein-rich ONS showed a boost in muscle mass, strength, and aerobic physical performance [19], and positive effects on e.g. activities of daily living (ADL) [20].

Interventions in NH are rare and those performed show limited or temporary effects. Collecting experiences from the participating older persons themselves may be key to understanding the reasons for this. In the Older Persons Exercise and Nutrition (OPEN) study, the effects on the functional status and independence of frail older persons in a NH of a combination of an STS exercise during routine care with a protein-rich ONS were evaluated [21]. The specific aim of this exploratory qualitative sub-study was to describe the older persons’ perceptions and experiences of being given the daily opportunity to perform the STS exercise and drink ONS.

## Methods

### Study design

This study was embedded in the OPEN study [21]; a two-arm randomized controlled trial performed in eight NH consisting of 62 dementia or somatic care units in the Stockholm County. The participants were randomized by NH units into an intervention group (IG) or a control group (CG). One hundred twenty participants were recruited, 60 participants in each group. Fifty-two persons constituted the intervention group who participated in a combined intervention of STS exercises (4 times a day) with intake of a protein-rich ONS (125 ml, 18 g protein, 300 kcal; Fortimel Compact Protein, Nutricia) twice daily for 12 weeks. Altogether 102 residents (86 ± 5 years, 62% females) completed the study (52 in the IG, 50 in the CG). No improvement in the physical function assessments was observed in the IG, although 21 participants with high adherence to the intervention, i.e. at least 40% compliance to the combined intervention, increased their fat free mass (2.12 kg (0.13, 4.26 IQR), *p* = 0.007) vs CG. Subgroup analyses indicated that high adherence to the combined intervention was also associated with maintained or improved physical function [22]. In this exploratory study, 23 selected residents were approached to participate in individual interviews 1–20 days after they each had their last day of intervention. The questions focused on their expectations from the intervention at enrollment (beforehand), and their experiences and perceptions during and after the interventions in relation to the two parts of the intervention. The interview guide developed for this study is provided as Additional File 1.

### Settings

Six NHs in two municipalities in Sweden were represented in the study. They were all small-scale units that applied a 2-shift work schedule for daytime/evening staff. All residents had a mini apartment including a space for simple cooking and dining, and a separate bathroom. The shared facilities in the NH included sitting room(s), kitchen & dining area, and some outdoor space. The management and rehabilitation quarters, which included a gym, were situated adjacent to the units. All NHs had appointed staff who each served as a designated main carer for a resident and therefore had in-depth knowledge about the resident, although all unit staff were part of the intervention. Since the study was conducted over 1.5 years, the interviews were completed over that period of time.

### Sampling and recruitment

Recruitment to this qualitative study included a combination of purposive sampling and theoretical sampling [23] to avoid selection bias. In the purposive sampling, staff suggested older persons who were viewed to have experience and awareness of the concept enough to be eligible to an interview (approx. 28 persons). Initially those who were not limited by their current health status or were indisposed for other reasons were interviewed. The addition of theoretical sampling meant the researcher approached participants based on specific characteristics, to reach broad nuance in the data. For example, in order to capture diverse experiences three ladies who regularly chose to train together in a small, shared sitting room were included. Recruitment continued until data saturation had been reached. As some respondents showed limited abilities to give nuanced responses, due to perseveration, dysphasia or memory loss, three more respondents´ than originally planned (see study protocol) were added to the study.

The participants, 7 men and 16 women (equivalent of the gender proportion in Swedish NH) aged between 76 and 96 years (mean 86 years, SD + 6.0), had lived at the NH long enough to be settled and included persons from both somatic (*n* = 10) and dementia (*n* = 13) units. Their Mini Mental State Evaluation scores ranged from 8 to 29 (mean 18, SD + 5.9) indicating that several had cognitive decline. They were all dependent on staff for one or several ADL domains.

### Data collection

One or, if there were any interruptions, two individual semi-structured audio-taped interviews were conducted by the first author in collaboration with authors HG & AMB.

All interviews entailed an introduction with reminders of the person’s participation in the OPEN concepts. The STS were illustrated by the interviewing researcher literary showing a few, and the ONS by showing an empty bottle of ONS. Both were used to prompt the persons´ memories before and if necessary, during the interview. The first author remained blinded to the person’s performance and adherence to the intervention. Using the semi-structured interview guide, the older persons were asked to describe their experiences and perceptions (i.e. thoughts and feelings) of the combined intervention, and their aspirations before and reflections after the intervention. Field notes, such as perceptions of the social and physical environment, were made during the visits [23]. Interviews were on average 45 min long.

### Data analysis

The interviews were transcribed verbatim and followed the constant comparative method suggested in Grounded theory [24]. Since the data collection was performed over more than 1 year the analytic process could be commenced and developed early in the project, consistent with theoretical sampling approaches. The interview transcripts were read through iteratively to become well acquainted with the data and gain insights into necessary additions to the interview guide. All transcripts were coded line by line and the underlined codes were then rephrased and described as a conceptual code. These codes were formulated in everyday language, as close as possible to the participants’ expressions.

Thereafter, all codes were taken together and systematically sorted into tentative categories and potential subcategories. In order to secure trustworthiness, the categories were then shared with and reflected upon by the research group. Lastly, the slightly revised categories and subcategories were analyzed to identify a potential overarching theme.

## Results

The results of the analysis consist of five main categories that each contain two to four subcategories (Table 1). Results are presented using examples and quotes to illustrate variety in the material and to increase trustworthiness for readers. Reference to residents’ quotes are abbreviated single letters, e.g. (F).

**Table 1:** The study findings

Overarching theme	Categories	Subcategories
A health concept with a social potential, as participants feel acknowledged and strong enough to help others	**1** Perceived hopes and expectations	Hope of improvement Low expectations were re-evaluated
	**2** Health-related driving forces	Internal driving forces Helping each other The importance of being acknowledged Becoming energized
	**3** Appreciated daily activity	A welcome change to daily routines Acknowledged the benefits of gaining strength Individualizing care for good balance
	**4** A concept easy to perform and integrate into daily life	Hardly noticeable, easily integrated into daily activities Low safety and spatial demands Flexibility on many levels
	**5** A beneficial health concept for all	Easily grasped health components “To the moon and back” Should be for all, and early on

### Perceived hopes and expectations

The analyses showed that the older persons thought that the combined intervention included aspects that brought ***hope of improvement*** into their everyday life, e.g. “*I thought to myself: Ten STS, how hard could it be?*” (A). The hopes were related to their life at the NH compared to their prior life, with individual aspirations such as” *[…*] *returning to the vigor I had before*” (A); and *“to be energized and stop my armchair sitting*” (B). Goals related to mobility were prominent, although reasons varied from “… *being able to get up and move about independently*” (C) to “*walk with a cane, so I can visit my family in Spain*” (D).

It appeared that initial ***low expectations were re-evaluated*** as the intervention proceeded: “*I am surprised that my legs gained so much strength just by adding some extra stand-ups during ADL. Being older, I thought there was not much to build on. But apparently …*” *(E*). Also, the expectations of the ONS, which were initially low, were exceeded over time. Descriptions ranged from “*unproblematic” (B)* to *“something of a treat”* (D), although some flavors were more popular than others.

Some remained more neutral in their judgements, such as this 97-year old: “*I chose to see the changes for what they are. No miracles, but a sense of improvement. I’m not getting any younger* “(C).

### Health-related driving forces

One of the major ***internal driving forces*** expressed was curiosity as to whether they could regain some strength, but also around the concept: “*I became curious as it was a new idea and a novel concept that I had never heard of before”* (F).

Some used their inner voice as a driving force in the training: *“I challenge myself by posing questions while I train: Can I do five more?*” (G). Others used their competitive thinking: “*I want to perform well and I love to compete... also with myself*” (D) or by finding some desire: “*I always did more, but only scored the compulsory ten as I wanted to do the rest just out of joy*” *(A*).

The analysis also underscored external driving forces such as the importance of ***helping each other.*** For example, supporting another project participant:” *I felt sorry for my ill friend, not being able to fully take part. Still, we have each other”* (C). The older persons were grateful for the support received during the intervention. One person received valuable support from her son, who adjusted the activities he did with his mother to her mobility at the time. Initially they re-planted flowers indoors, then did some outdoor gardening, and later took walks outside.

Although most had chosen to perform the health concept individually and within the space of his or her own NH apartment, some reflected on the potential of group interventions at the unit: *“It would have to be led by someone enthusiastic. Not all staff are suited for it*” (A). A few described how they had taken on a leader role to encourage their companions to move:” *I had the opportunity to guide others - a really nice experience*!” (A).

Lastly, some needed *more* support from others:” *Reminders and encouragements are what I need. I am even unsure of the extent to which I adhered to the intervention”* (H). Lack of sufficient support was also experienced:” *They were supposed to serve me drinks twice daily. But I had to remind them. Frustrating!*” (F).

Another driving force related to ***the importance of being acknowledged*** for their adherence and ambitious efforts. One participant proudly reported that she had “[ …] *independently performed the STS and taken the ONS perfectly. With an immaculately filled flowchart, staff could confirm*” (B).

Others pointed out the gains perceived by working closer with the staff. Several commented on their nice collaboration during the intervention, although they knew the staff were obliged to support them in performing the daily STS and taking the ONS. In several instances, they reminded one another about doing the STS during daily life activities, to refill the fridge or document progress. Some older persons were disappointed that the staff collected their flowcharts, as they wanted to show family and friends their progress (C + F).

Staff seemed to acknowledge the older persons’ individual needs, negotiating personalized set-ups for the combined intervention. At one unit, the STS was provided for one person prior to the intake of the ONS, which became a “*reward he had deserved*”. Someone else drank the ONS before performing the STS to gain energy, while she had her second ONS as an “*avec*” with her evening coffee.

One of the driving forces in the intervention was the feeling of ***becoming energized.*** “*I joined the project with few expectations, but of course I had to continue. The drinks … wow, they were perfect! Good size, pleasant taste and nice texture mm*” (C). *“I feel SO much more energetic compared to my earlier limitations”* (A)*.* This sense of energy was a continuous driving force that the older persons used to try to remind themselves to keep going while counting the STS. One participant added a nice view:” *I do them by my window to gaze out at nature. To find something to focus on*” (A).

### Appreciated daily activity

The analyses showed that the combined intervention was **a** ***welcome change to the daily routines***. Initially, it seemed somewhat challenging to integrate into the day:” *[…*] *it depended on the abilities of the staff to distribute the ONS regularly, it was messy at first*” (I). However, after the intervention, some even chose to implement the health regimen as an addition to their daily routines e.g. during morning wake-up: “[ …] *just to do a few STS to get myself going*” (F). Similarly, two persons described taking their ONS before getting out of bed (J + K). One argued: “[ …] *to fill up my energy quota*” (K). Also, the ONS was more appreciated when served chilled, which meant that several made the effort to walk to their fridge to fetch the flavor they wanted:” *I have changed my routines and now get up and walk, also to the TV to change the channels*” (C).

Findings also showed that the older persons ***acknowledged the benefits of gaining strength***. A sentence that was often heard was: “*I feel benefits from being strong in my new everyday life*” (L). Some described how their increased strength allowed new routines:” *I now make my bed daily and … well, I have set the rule for myself to go outdoors daily”* (G).

Several of the older persons wanted to continue their new, more active life. Descriptions of attempts to maintain the STS/ONS concept after the study had finished concerned ***individualizing care for good balance***:” *I want to find balance in my daily routines; being active without getting sore muscles, which I dislike*” (L). Some also expressed concern about not getting the ONS any more. Someone asked the researcher:” *Is there a possibility to get them at times in life when energy might run low?”* (C).

### A concept easy to perform and integrate into daily life

The findings also illustrated that the intervention was ***hardly noticeable, easily integrated into daily activities.*** Several older persons indicated that they liked the simplicity of the routine: “*It was so easy to integrate. I noticed that I still have some learning potential in me”* (C). Several described how they had integrated the STS exercise into other daily activities: “*I stand up and pull my trousers up without any support now*” (M). “*I don’t see the harm in it, the sacrifice of doing the STS is a small one*” (C). Similarly, the intake of the ONS was described as being easy to add to the activities of the day: “*… really uncomplicated and easy to deal with*” (F), “*… the staff delivered the ONS to us*” (M). One person described their drill:” *I just grab hold of it and let it pour down my throat in one serving. No big deal!” (*A). Others reflected on the whole concept: *“I didn’t regard any of it as a strain. In fact, it was almost too easy. I once said: This is easy to the point of being silly” (N).*

Another aspect of the health concept being easy concerned the high level of applicability due to ***low safety and spatial demands,*** i.e. it could be easily and safely performed***.*** In both the field notes and interviews, the persons’ eagerness to show their environmental adaptations to the STS were evident. Most chose their favorite chair, others chose a chair because of its armrests, its sturdiness or even that it was odd: *“I chose this chair because it stands out, and seeing it reminds me to do the STS*” (A). Some cited safety reasons: “*I either use a chair at my dinner table, or get up from my armchair with my roller locked securely in front of me*” (C) or: “*I have found the perfect way for me to perform them (pointing to the footboard on her bed that she used as a support to stand up)*” (O). Similarly, a few described using the railings in the corridors of the NH for STS support.

The data analysis also described the concept as ***flexible on many levels.*** Some described the ONS as small enough for one serving, while others used a straw or a glass with measurements on it: “*I used the glass to monitor my intake. I drank it in about one hour*” (P). Others emphasized the flexibility of the STS: “*It is flexible, so I increased my effort over time, to give all my muscles their share. When I started I had my arms straight out like a lever when I pushed up. Now I have my arms folded over my chest or even raised above my head”* (A). Another participant had her own approach to the STS exercise: “*For me it works best to get them done all at the same time, so I do all 40 STS once a day* (F)*.* A few described needing the concept to be flexible:” *I can occasionally, but not all the time. That would be too tiresome*” (Q).

### A beneficial health concept for all

Descriptions of the ***easily grasped health components*** of the combined intervention were very common: *“I think we added STS exercises in all the everyday activities. Going to the bathroom, washing at the sink, dressing … so the legs got their share of training. And it still feels fine”* (F). The same man described his increased mobility: “*I have started taking walks in all the corridors. I stroll around and sit on different seats along the way*” (F).

Most people reflected on the benefits of the health concept. A few persons initially described being unsure if any changes had occurred although eventually stated: *“Well, I’m sure it does some good as I feel more alert. But it’s hard to tell since the dosage was quite limited”* (C). Likewise, some mentioned now having “*a good feeling*”, or “*a more positive outlook on life”,* and others being more limber:” *Now, I can even get up from extremely low heights without support. So improvements have been noticed, definitely” (D).* “*I was at my son’s the other day and I could easily get up from that low toilet of his. I was beyond pleased*” (G).

One person even described impressive improvements: “*I could walk* ***to the moon and back.*** *Go out on my own again – without my cane! I am happier and more energized. You see? Just start whenever. It is never too late”* (A). This participant had noticed that the ONS was high in protein and shared her thoughts:” *I think a bit differently now,* e.g. *about this bread I eat. I have started buying rye bread that contains more protein. I have it here, you see? Because the bread that I am served here only consists of empty air, as I see it”* (A).

The last subcategory in the findings touched upon the older persons’ recommendations for future health interventions. They re-iterated the view that the concept ***should be for all, and early on***. One lady illustrated what several concluded: *“As I see it, it would be most practical to include all of us at the unit. No problem*” (M). Several commented that they would have benefited from this on admission to the NH: “*I sincerely think that this is a good concept to offer anyone already on arrival to the NH”* (A). Some arguments were: “*Especially if you consider the fact that you become steadier and improve balance*” (C).

Interestingly, about half of the older persons spontaneously told the interviewer that they would definitely participate again, given the opportunity. One emphasized the influence the concept had on her mood: *“I loved that it was … a mixture. That I got energy from the ONS too. I was a little bit down at the time. Not depressed, but I couldn’t walk outside independently. After having done the intervention, I was much more able”* (A).

Final words from some of the older persons concerned their own choice to continue: *“I have continued training. I believe it is good to hang in there and keep it up*” (M), and the insightful comment from a participant: “*I have continued, for the sake of my own health*” (C).

The last step of the analysis included the identification of an overall theme. In this study, the older persons’ experiences of the combined intervention was viewed as ***A health concept with a social potential, as participants feel acknowledged and strong enough to help others.***

## Discussion

As illustrated above, the STS/ONS concept had various perceived health benefits. The study evoked hopes of improvement and displayed several health-related driving forces. It also seemed to be an appreciated activity in their daily lives, as the concept was viewed easy to perform and integrate. Many perceived the health benefits as positive and repeatedly suggested that the STS/ONS would be a beneficial health concept for all.

### The importance of taking older persons’ competencies and personal driving forces into account

An important factor in the health concept in the OPEN study was its broad introduction to staff beforehand. This enabled staff to reflect on how to feasibly integrate the combined physical exercise and ONS into the older persons’ daily lives. At first glance, the concept seemed fixed, but then proved to be flexible and could be adapted to each older person’s daily life and unique needs.

In our study, most participants faced diverse challenges that needed to be addressed. Thus, the staff who knew the older persons’ individual preferences were encouraged to provide individually tailored support. The ambition was to encourage staff to adapt and individualize the daily care so that the aspects of the intervention could be integrated. They were encouraged to take on a salutogenic, i.e. a health-oriented, approach rather than the more common pathogenic focus where dysfunctions are given more attention. The health-oriented approach considers aspects in the persons’ lives that are still meaningful to them [25], while a pathogenic focus on dysfunctions has been shown to undermine a person’s sense of competence [26] and conflict with their health aspirations. Findings from our study, and those by Phinney et al. [27], show that older NH residents are motivated to remain active and independent.

A core foundation in this project is the person-centered approach. Such an approach calls for interaction between the older person receiving the service and the staff who provide it [7]. Decisions made in the intervention were based on a partnership between the older person and the staff with respect to the older person’s own competencies and personal driving forces. This concurs with the Swedish Social Service Act to provide care to meet each person’s individual needs [8] and the Swedish NBHW statement that the care of older people must preserve their right to live with dignity [6].

### Applying a power-sharing care stance can result in increased sense of self worth

Interestingly, the study seems to have resulted in a power equity between the older persons and the staff. For example, participants and staff reminded each other about the intervention, such as the plan or to fill in the flowchart. Some of the perceived health benefits are believed to be a result of staff applying a power-sharing stance towards the residents at the NH. Signs of trust in the older persons’ abilities to change their daily habits can increase their sense of self-worth, as shown in a study by Arola et al. [28]. This also seems in line with findings from other NH interventions in which enhanced commitment to daily activities was observed when activities were selected out of personal interest [29] or personal aspirations [30].

Another illustration of trust and, thus shared power, is that several participants had their own flowcharts for each week of the intervention. This can serve as a memory strategy with a visible reminder and may increase adherence through a sense of empowerment [31].

Although each older person was in charge of their own process, it is interesting to identify some mixed messages. Even if they felt empowered by the researchers and the staff, they could also feel excluded when the staff replaced the completed flowchart with a new weekly chart. This was evidence of unclear boundaries in ownership between the staff and the older person; an example of this was a resident who wished to show the flowcharts to visitors. This confirms that implicit or explicit rules can suppress the resident’s autonomy, as previously described [10]. These frustrations indicate that at least some of the older persons were empowered by the project, which is rarely reported in the literature. Instead, power imbalances after admission to institutions are commonplace [29].

Perhaps the empowered older persons induced a power-sharing stance in staff through the staff slowly convincing the older persons of their abilities. Such increased power sharing might then, in turn, further strengthen the older person’s sense of self-worth. This is in line with the reasoning of Tak et al. [32] that older persons in NH should be included in activity planning and receive adequate assistance to engage in activities meaningful to them.

### Comprehensible, flexible interventions strengthen participant adherence

The STS/ONS health concept was simple and easy to perform on many levels. The physical exercise consisted only of STS movements and the 125 ml ONS, albeit in a range of flavors, was always the same format and texture. Moreover, the intervention had a built-in adaptability in that the exercise and the ONS were single components in their respective parts of the health concept.

In a meta-analysis, Benjamin et al. [33] identified three barriers to performing physical exercise in long-term care facilities, such as NH. The first barrier was the health status of the participant and his or her ability to manage the complexity of the exercise. The second was environmental, such as lack of space to perform the physical activity. The third was related to economic constraints, such as lack of staff to support the older persons.

The STS exercise is adaptable to the health status of older persons, as long as they can get up from a chair, either with or without using their arms. Also, this study demands little physical space. Even if the older persons have smaller rooms, space for a chair is all the exercise requires. It also seems that the older persons’ dependency on staff was slightly less since they adapted their own ways to perform the STS. Further, the older persons illustrated that the exercise could be well integrated into daily life activities and routines, such as hygiene and dressing. Hence, the combined intervention seems to be a promising way to optimize adherence.

### Towards a nursing home environment that also takes social needs into account

The primary outcome in OPEN intervention analyses; i.e. the capacity to rise from a chair, measures a combined effect of mainly thigh strength and balance, and is a highly important capacity for independency of older people. This resonate with the perceptions found here, as several residents witnessed signs of improved perceived health, such as feeling energized from taking ONS, feeling steadier with improved balance, more mobile, having the ability to perform the STS exercises without support, and there also seems to be mental health benefits. Tentatively, one might ask whether the perceived improvement in physical functions from this ‘health intervention’ also contributed, in some cases, to an improved mental status. Earlier studies have convincingly related improved physical function with improved mood [16, 34].

When an older person’s mood changes, the response from others might also change. Family and friends might perceive the older person differently if they see them being included, respected and treated equally. The opposite has also been shown to be a health hazard. People who are not included and lack interaction are at risk of occupational disengagement, which may even lead to occupational deprivation [35]. Our findings indicate that participants valued supporting each other, empowering other residents and contributing to a more meaningful day. Parallels with our findings can be drawn from a study by Schmidt et al. [36], which showed how older persons identified social activities as crucial facilitators to eventually mobilizing the strength to perform physical activities. Lorenz et al. [16] also found similar indications to ours, where interventions need to be experienced as meaningful to be viewed positively. Likewise, a neuro-psychological study of persons with Alzheimer’s disease found that successful interventions can take place in less controlled, real-world contexts, if they directly focus on daily life situations and involve the target persons. Van der Linden & Juillerat [30] argue that their approach was successful because it allowed those with dementia to maximize their abilities, state specific preferences and needs, and make minor intervention-related adjustments. Their findings are fully in line with that described by the older persons in this study, who also had wishes and health aspirations. This study also acknowledges their perceived benefits of collaborating with staff and having the concept as something that gave meaning to their day.

If the passive lifestyle commonly associated with NH life becomes more active, the older persons’ mood and, therefore, sense of health might improve. Potentially, passivity might be replaced with an easily applied activity supported by an oral supplement, such as the STS/ONS concept. This is strengthened by the quantitative OPEN health-economics data found using the Resource utilization in dementia (RUD) instrument [37] that identified significant decrease in caregiver time in the intervention group with 15.7 min/day (*p* = 0.04), which might for example even out costs related to the ONS drinks.

### Methodological considerations

For qualitative analysis, the data from the 23 older persons proved to be rich enough to reach data saturation, i.e. a state where answers started re-occurring and new unique material ceased to appear [23]. To further secure the trustworthiness of the study, some fellow researchers reflected on the findings at an early stage of the analysis [38] while the rest of the team – experienced researchers in the field of geriatrics - confirmed its credibility [24].

As staff played a role in fulfilment of the interventions, they too were interviewed for a forthcoming qualitative publication from the OPEN study.

## Conclusion

This qualitative study on the implementation of a health concept in NH combining daily STS exercises with ONS intake could be easily implemented in daily routines adapted to each resident’s capabilities. The concept showed perceived benefits on various health aspects, such as feeling energized and stronger. Moreover, internal and external motivators were related to the individual empowerment and sense of competence of the participants, and collaboration with staff. These aspects likely improve the adherence to such health interventions.

Health concepts, such as the OPEN concept, could potentially provide older persons with hope of improvement and contribute to a more meaningful day. They may even be of interest to older persons in a NH in the fragile and vulnerable end-of-life period.

## Supplementary information

**Additional file 1.** The semi structured interview guide developed for the study

## Data Availability

The datasets used and/or analysed during the current study are available from the corresponding author on reasonable request.

## Abbreviations

OPEN: Older Person’s Exercise and Nutrition
STS: Sit-to-stand
ONS: Oral nutritional supplementation
NH: Nursing home
ADL: Activities of daily living
