# Supplementary material for: A health concept with a social potential: an interview study with nursing home residents
Source: BMC Geriatr. 2020 Sep 4;20:324. doi: 10.1186/s12877-020-01731-4 (PMC7487501; doi:10.1186/s12877-020-01731-4)
Supplement: Supplementary file 1 — Additional file 1. The semi structured interview guide developed for the study [file 12877_2020_1731_MOESM1_ESM.docx]

**Additional File 1**

The semi structured interview guide developed for the study

1. *Can I ask you to describe how you have experienced participating in this OPEN study concept?*
2. *Could you tell me your thoughts and your feelings towards doing the physical exercise sit-to-stand?*
3. *Could you tell me your thoughts and your feelings towards the nutritional supplements you received during the OPEN project period?*
4. *Is there something else you have in mind in relation to the nutritional supplements?*
5. *Before the OPEN concept began. What were your aspirations and thoughts around the benefits it might have?*
6. *What does your life look like now- after the project period – in relation to supplement drink intake and training?*
7. *Do you have any thoughts for us on how this STS/ONS concept could be developed differently?*
